# Adult Malrotation With Midgut Volvulus: A Case Requiring Temporary Abdominal Closure and Second-Look Operation

**DOI:** 10.7759/cureus.105171

**Published:** 2026-03-13

**Authors:** Tyler A Finkenthal, Abhiram Kondajji

**Affiliations:** 1 General Surgery, Cleveland Clinic - South Pointe Hospital, Warrensville Heights, USA

**Keywords:** adult intestinal malrotation, congenital disease, discontinuity, emergency exploratory laparotomy, emergent general surgery, intestinal ischemia, ladd's procedure, malrotation, midgut malrotation, temporary abdominal closure

## Abstract

Malrotation is a rotational anomaly of the embryonic bowel, which can be complicated by midgut volvulus. This phenomenon is typically seen in the pediatric population. Presentation in adults is rare and can present diagnostic challenges that delay diagnosis and time from presentation to definitive management in the operating room. In this case, a 43-year-old female with a past medical history of anxiety and no prior abdominal surgery presented to the emergency department with four days of abdominal pain and obstipation. She was ultimately determined to have malrotation with midgut volvulus based on intraoperative findings. There are other case reports involving adult malrotation in the current body of literature. This case is unique as the patient presented with necrotic small bowel requiring resection, temporary abdominal closure, and a second-look operation to evaluate intestinal viability and restore continuity of the bowel.

## Introduction

Intestinal malrotation arises from the arrest of normal embryologic gut rotation between the fourth and tenth weeks of gestation. During this period, the midgut typically elongates, herniates into the extraembryonic coelom, and undergoes 270 degrees of counterclockwise rotation around the axis of the superior mesenteric artery before returning to the abdominal cavity [1]. This normal process positions the duodenojejunal junction to the left of the midline, where it is fixed to the retroperitoneum by the ligament of Treitz. The cecum is then gradually fixed in the lower right quadrant. This normal rotation and fixation create a wide-based mesentery that helps prevent volvulus [2].

Several types of rotational anomalies have been described, including malrotation, nonrotation, and reverse rotation. In malrotation, the duodenojejunal limb assumes a position of nonrotation, while the cecocolic limb undergoes partial rotation. The cecum is often located in the mid-upper abdomen and is fixed to the right lateral abdominal wall by Ladd bands. The short mesentery of the displaced midgut forms a narrow pedicle around the superior mesenteric artery, predisposing it to rotation, which can result in volvulus and subsequent midgut ischemia [3].

The incidence of midgut malrotation is approximately 1 in 6,000 live births, with nonrotation being the most common anomaly, observed in approximately 2 per 1,000 contrast-enhanced upper gastrointestinal studies [4]. The most severe presentation of gastrointestinal rotational anomalies is volvulus. Between 64% and 80% of malrotation cases present within the first month of life, and 90% present within the first year. Only 0.2% to 0.5% of cases present in adulthood, and of those, approximately 15% involve midgut volvulus [5]. Due to the rarity of this diagnosis in adults, identification is often delayed, leading to increased morbidity and mortality. In a study by Durkin et al., none of the adult patients were correctly diagnosed at the time of symptom onset, despite 70% experiencing chronic symptoms for six months or longer [6].

This case report discusses a 43-year-old female who presented with malrotation complicated by midgut volvulus. This case not only discusses a rare pathology in the adult population but presented the management challenge of necrotic bowel requiring temporary closure and a second-look operation.

## Case presentation

A 43-year-old female with a past medical history of anxiety and no prior surgical history presented to the emergency department with a four-day history of progressively worsening abdominal pain, accompanied by nausea, vomiting, and obstipation. She also reported anuria over the preceding 24 hours and a cough, but denied any other symptoms at the time of presentation. Prior to presentation, the patient reported an absence of prior chronic gastrointestinal symptoms.

**Figure 1:**
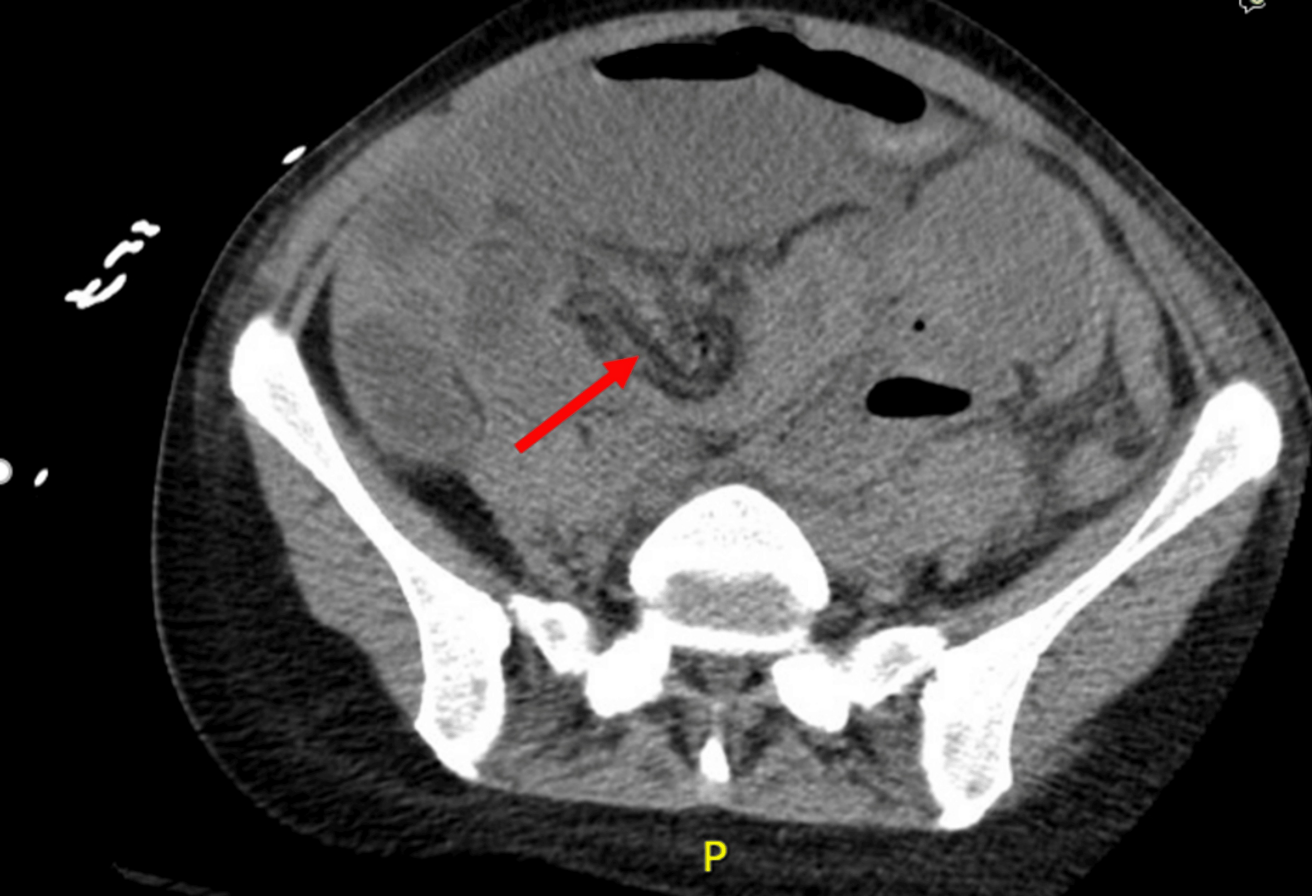
CT abdomen and pelvis without IV contrast (axial) Axial view of the CT abdomen and pelvis revealing swirling and edema of the mesentery (arrow). Also seen is dilation of the small bowel, which is notably seen in the right abdomen of this axial image.

**Figure 2:**
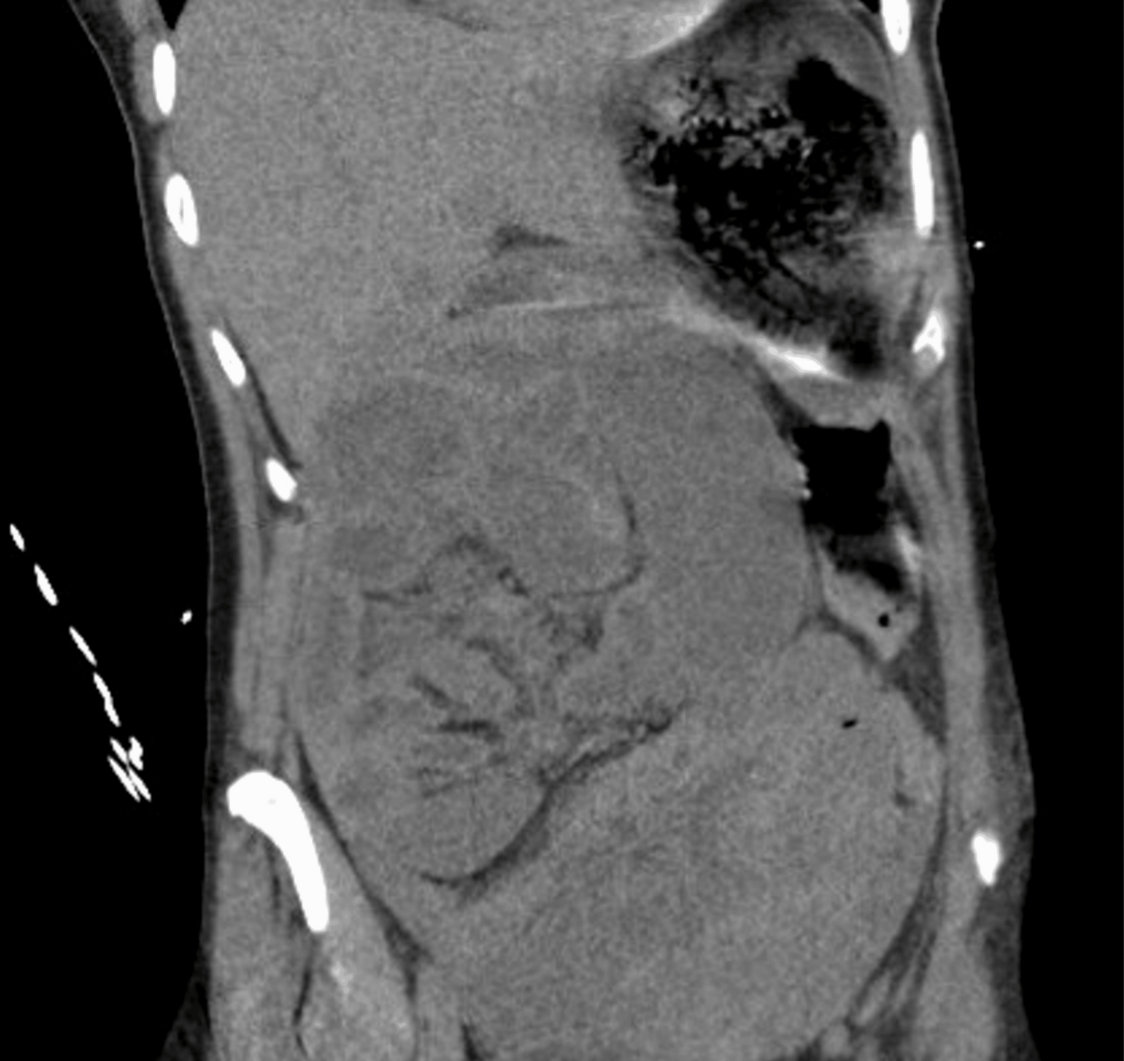
CT abdomen and pelvis without IV contrast (coronal) Coronal view of the CT abdomen and pelvis, revealing dilated small bowel all on the right side of the abdomen, and a lack of cecum in the right lower quadrant of the abdomen.

Initial emergency department work-up included laboratory testing and imaging. Significant laboratory findings were consistent with severe acute kidney injury (AKI), leukocytosis, anion gap metabolic acidosis, hyponatremia, hyperkalemia, and elevated lipase, with a normal lactate level (Tables 1-3). A CT scan of the abdomen and pelvis without intravenous contrast revealed dilated loops of small bowel measuring up to 4.2 cm, with associated edema and swirling of the mesentery, concerning for small bowel obstruction with volvulus (Figures 1, 2). General surgery was consulted. 

**Table 1:** CBC with differential at presentation CBC: Complete blood count; WBC: White blood cells; RBC: Red blood cells; MCV: Mean corpuscular volume; RDW: Red blood cell distribution width; MCHC: Mean corpuscular hemoglobin concentration; MCH: Mean corpuscular hemoglobin.

CBC With Differential at Presentation	Patient’s Results	Normal Range
WBC	22.46 K/uL	3.70-11.00 K/uL
RBC	3.76 m/uL	3.90-5.20 m/uL
Hemoglobin	11.0 gm/dL	11.5-15.5 gm/dL
Hematocrit	30.5%	36.0%-46.0%
MCV	81.1 fL	80.0-100.0 fL
RDW	12.9%	11.5%-15.0%
MCHC	36.1 g/dL	30.5-36.0 g/dL
MCH	29.3 pg	26.0-34.0 pg
Platelet Count	352 k/uL	150-400 k/uL

**Table 2:** Basic metabolic panel at presentation. BUN: Blood urea nitrogen; CO_2_: Bicarbonate

Basic Metabolic Panel at Presentation	Patient’s Results	Normal Range
Glucose	109 mg/dL	74-99 mg/dL
BUN	115 mg/dL	7-21 mg/dL
Creatinine	7.34 mg/dL	0.58-0.96 mg/dL
Sodium	122 mmol/L	136-144 mmol/L
Potassium	5.8 mmol/L	3.7-5.1 mmol/L
Chloride	74 mmol/L	98-107 mmol/L
CO2	20 mmol/L	22-30 mmol/L

**Table 3:** Other laboratory studies at presentation

Other Laboratory Studies at Presentation	Patient’s Results	Normal Range
Lipase	762 U/L	16-61 U/L
Magnesium	3.2 mg/dL	1.7-2.3 mg/dL
Lactate	1.7 mmol/L	0.5-2.0 mmol/L

On initial physical examination, the patient was tachycardic to 120 beats per minute with a stable mean arterial pressure. Her abdomen was distended, with severe tenderness to palpation, guarding, and rigidity, raising concern for diffuse peritonitis. Broad-spectrum antibiotics (levofloxacin and metronidazole) were initiated. A nasogastric tube (NGT) was placed for low intermittent wall suction (LIWS), deep vein thrombosis (DVT) prophylaxis with subcutaneous heparin was administered, and fluid resuscitation was initiated while preparations were made for exploratory laparotomy.

The patient was taken to the operating room on the day of presentation. A generous midline incision was made and carried down to the peritoneal cavity. Intraoperatively, a large band was noted crossing the midline, originating from the right upper lateral abdomen, and necrotic small bowel was identified in the pelvis. The necrotic bowel was delivered and detorsed. The band was noted to be tethering the right colon and cecum just to the left of the duodenojejunal junction, consistent with a Ladd’s band, which was divided using cautery.

The small bowel was traced proximally, and 110 cm of viable jejunum was identified proximal to the area of necrosis. The bowel was transected at this point using an Endo GIA 60 mm stapler (tan load). The mesentery of the necrotic bowel was taken with a LigaSure device until approximately 50 cm proximal to the ileocecal valve. The viability of the ileum appeared questionable, but frankly not ischemic. The small bowel was transected again at this juncture. An appendectomy was also performed. The abdomen was irrigated with saline. At this point, the patient began exhibiting signs of coagulopathy with diffuse oozing from surgical planes. Given exhausted physiology and the uncertain viability of the ileum, a decision was made to perform a temporary abdominal closure and leave the patient in discontinuity.

A sterile fenestrated isolation bag was placed around the intestinal contents and covered with a scannable towel. Two 10 mm flat drains were placed at the wound edges and brought out through the inferior portion of the abdominal incision. An Ioban dressing was applied over the entire abdomen. The patient was transferred to the intensive care unit (ICU) for continued medical management with plans for a second-look operation.

Two days later, the patient returned to the operating room for re-exploration and either restoration of bowel continuity or creation of an ostomy. The abdomen was irrigated, and the remaining Ladd’s bands were removed. The ileum was found to be viable. An anti-peristaltic side-to-side jejunoileal anastomosis was created using two firings of an Endo GIA 60 mm and one 30 mm stapler (tan loads). The common enterotomy was closed with 2-0 polydioxanone (PDS) suture and reinforced with a Lembert 2-0 silk suture. The anastomosis was tension-free, well vascularized, and leak-free. The mesenteric defect was closed. After confirming the appropriate positioning of the bowel, with the colon in the left abdomen and the small bowel in the right abdomen, the abdomen was irrigated with Irrisept. The midline fascia was closed, irrigated again, and the skin was closed. The patient returned to the ICU intubated postoperatively.

Postoperatively, the patient’s respiratory status, complicated by right lower lobe pneumonia, was managed by the ICU team, and renal function was managed by nephrology. She was extubated on postoperative day one and started on peripheral parenteral nutrition (PPN). She resumed bowel function by postoperative day three and was transferred to a regular nursing floor. A liquid diet was initiated on postoperative day five and advanced to a soft diet by postoperative day eight, at which time PPN was discontinued. She was discharged to an acute rehabilitation facility on postoperative day 10.

At outpatient follow-up one and a half months after surgery, the patient reported doing well. She was tolerating a regular diet, having normal bowel function, and healing without complications.

## Discussion

Malrotation is a congenital anomaly of the rotation and fixation of the midgut during embryologic development. It is characterized by incomplete intestinal rotation, which causes the cecum to remain in the epigastrium. In this position, Ladd’s bands may develop and cross over the second portion of the duodenum. These bands can cause duodenal obstruction, while the abnormally narrow mesenteric pedicle, confined to the epigastrium, increases the risk of volvulus. A volvulus in this setting can compromise branches of the superior mesenteric artery that supply the midgut, leading to intestinal ischemia and a surgical emergency [3].

The case presented illustrates that malrotation can manifest with acute and severe complications, requiring emergent surgical intervention, bowel resection, and extended hospitalization. Due to its rarity in adults, malrotation is frequently misdiagnosed or not considered. Only 10-15% of adults with malrotation present with acute midgut volvulus [7], as seen in this case. Typical features of acute midgut volvulus include sudden-onset severe abdominal pain, vomiting, and hemodynamic instability. Hematemesis or hematochezia may suggest bowel ischemia or necrosis. On physical examination, findings can include abdominal distension and diffuse tenderness, with or without signs of peritonitis [8]. The patient described in this case exhibited a majority of these acute symptoms. Given the rarity of adult malrotation with midgut volvulus, this diagnosis was not high on the initial differential. Her initial clinical picture raised high suspicion for the need to proceed with urgent operative intervention.

While it is important to recognize the acute presentation of midgut volvulus, it is equally crucial to consider the chronic symptoms associated with malrotation. Approximately 88% of adults with malrotation present with chronic or intermittent symptoms, which may include abdominal pain, vomiting, obstipation, weight loss, food intolerance, malabsorption, chronic diarrhea, pancreatitis, and motility disorders [9,10]. Some patients may remain asymptomatic, with the diagnosis made incidentally on imaging studies. In cases of acute presentation, as in this patient, identifying a prior history of chronic gastrointestinal symptoms may raise clinical suspicion for malrotation with volvulus.

Lactate, often measured in peritonitis workups, was normal in this case despite intraoperative necrotic small bowel. This is a clinical discrepancy, as lactate signals tissue hypoperfusion. Guidelines note that normal serum lactate does not exclude acute mesenteric ischemia as the liver can clear substantial L-lactate from the porto-mesenteric circulation [11]. Midgut volvulus twists the mesentery, frequently obstructing the superior mesenteric vein and causing congestion [12]. This may impede lactate return to systemic circulation, which would be detectable peripherally.

CT of the abdomen is one of the most commonly used modalities for evaluating abdominal pain in adults. Radiologic findings suggestive of malrotation include a duodenum that fails to cross the midline, a cecum located in the left abdomen, and reversal of the normal relationship between the superior mesenteric vein (SMV) and artery (SMA), with the SMV abnormally positioned to the left of the SMA [13]. The presence of the "whirlpool sign" on imaging may indicate volvulus. In non-emergent cases, an upper gastrointestinal (UGI) series remains the gold standard for diagnosing malrotation, as it allows for optimal visualization of the duodenum. Other imaging modalities, such as ultrasound and plain radiography, may reveal signs of acute intra-abdominal pathology but are generally insufficient to exclude malrotation [14].

The definitive treatment for malrotation with volvulus is the Ladd procedure. This includes detorsion of the volvulus, division of Ladd’s bands (fibrous adhesions between the colon and duodenum), and widening of the mesenteric base by incising the peritoneum over the SMA. An appendectomy is typically performed to prevent future diagnostic confusion, given the repositioning of the cecum. The bowel is then arranged with the small intestine placed on the right and the colon on the left to minimize the risk of recurrent volvulus [15].

Surgical correction may also be considered electively in patients with malrotation who are asymptomatic or present with chronic symptoms, in an effort to reduce the risk of future volvulus. Studies comparing laparoscopic and open approaches to the Ladd procedure have yielded mixed results. Some evidence suggests that laparoscopic techniques result in suboptimal mesenteric widening [16], while other retrospective analyses have found no significant difference between laparoscopic and open approaches in preventing recurrence of volvulus [17].

In this case, the application of temporary abdominal closure was an essential component of the surgical strategy. It is a valuable technique in both traumatic and non-traumatic abdominal surgeries, particularly when definitive closure is contraindicated due to patient physiology or intraoperative findings. Common indications in non-trauma settings include abbreviated laparotomy due to severe physiologic derangement, the need for a deferred intestinal anastomosis or a planned second look for intestinal ischemia, a persistent source of peritonitis with failure of source control, or extensive visceral edema and a concern for abdominal compartment syndrome [18]. In our patient, temporary abdominal closure was utilized for two of these indications, including a planned second look for intestinal ischemia and the need for an abbreviated laparotomy due to severe physiologic derangement. This approach permitted ongoing resuscitation, correction of coagulopathy, and time for the bowel of uncertain viability to declare itself, all within the controlled environment of the intensive care unit.

Guidelines recommend negative pressure wound therapy with continuous fascial traction as the preferred method for temporary abdominal closure [18]. Additionally, for cases with suspected or evolving intestinal ischemia, re-laparotomy is recommended within 24-48 hours of the index operation. Earlier re-exploration may be warranted if the patient’s hemodynamic status deteriorates or fails to improve [18]. In this case, the patient demonstrated clinical and laboratory improvement over the initial postoperative period, allowing for re-laparotomy at approximately 48 hours, in line with best practice recommendations.

## Conclusions

This case highlights the importance of including intestinal malrotation in the differential diagnosis of acute abdominal pain in adults. Timely recognition and surgical intervention are essential to preventing life-threatening complications such as midgut volvulus and bowel ischemia. Maintaining a high index of clinical suspicion for malrotation in patients presenting with chronic postprandial gastrointestinal symptoms may aid in preventing acute presentations. The Ladd procedure remains a safe and effective treatment in both acute and elective settings. While it has traditionally been the mainstay of therapy in pediatric populations, it is equally relevant in adult patients. As illustrated in our case, the use of temporary abdominal closure followed by a second-look laparotomy is a viable strategy to preserve viable bowel and minimize the risk of complications such as short bowel syndrome. In summary, early diagnosis and appropriate surgical management are crucial to decreasing the severe outcomes that can be seen in adult malrotation.
